# LASSO model selection with post-processing for a genome-wide association study data set

**DOI:** 10.1186/1753-6561-5-S9-S24

**Published:** 2011-11-29

**Authors:** Allan J Motyer, Chris McKendry, Sally Galbraith, Susan R Wilson

**Affiliations:** 1 Prince of Wales Clinical School, University of New South Wales, New South Wales 2052, Australia; 2 School of Mathematics and Statistics, University of New South Wales, New South Wales 2052, Australia

## Abstract

Model selection procedures for simultaneous analysis of all single-nucleotide polymorphisms in genome-wide association studies are most suitable for making full use of the data for a complex disease study. In this paper we consider a penalized regression using the LASSO procedure and show that post-processing of the penalized-regression results with subsequent stepwise selection may lead to improved identification of causal single-nucleotide polymorphisms.

## Background

For a complex disease with many causal genetic factors an analysis that simultaneously takes into account the effect of all single-nucleotide polymorphisms (SNPs) is preferable to one in which each SNP is considered separately. In particular, a simultaneous analysis of all SNPs will be able to identify those SNPs that have a strong joint effect in the presence of other causative SNPs but are not necessarily identifiable in a single-marker analysis because the effect of the SNP acting alone is not strong enough to be detected. The simultaneous analysis of all SNPs in genome-wide association studies (GWAS) for a complex disease is under active consideration; see, for example, [1-4].

We approach the problem of model selection (i.e., identification of the SNPs that are associated with the phenotype) by making use of penalized-likelihood methods [5], in particular, the LASSO (least absolute shrinkage and selection operator) procedure [6], which is briefly described in the Genetic Analysis Workshop 17 (GAW17) background paper on machine learning methods [7]. We consider a two-step model selection procedure for simultaneous analysis of all SNPs in GWAS. The first step is to apply the LASSO method for penalized-likelihood variable selection to identify a set of SNPs for further consideration. Setting the LASSO tuning parameter is somewhat arbitrary, so we set these parameters to include a relatively large number of SNPs and used a second step to refine the model by using a traditional variable selection method with the candidate SNPs selected in the first step.

We also assessed the performance of the LASSO with resample model averaging. This assessment is able to show whether SNPs in our model are the most often selected SNPs when using bootstrap samples, which suggests model stability; that is, the model is not likely to change with small changes in the data.

## Methods

Our analysis was carried out for the unrelated individuals in the GAW17 data set. We used phenotype replicate 1 for model selection and considered the quantitative trait Q1 as the response variable. Our approach is suited only to common variants. We filtered out SNPs with a minor allele frequency (MAF) less than 0.01. We identified SNPs for inclusion in a multivariate linear model with additive SNP effects and identified non-SNP covariates with the two-stage procedure already described. In the first stage we carried out the LASSO procedure using the glmnet package [8] in the R software (http://www.R-project.org). The tuning parameter *λ* was determined using a 10-fold cross-validation (CV). The non-SNP covariates (Sex, Age, and Smoke) were included as unpenalized terms. All covariates were standardized before running the LASSO procedure.

The second stage involves performing a stepwise selection with the SNPs selected in the first stage and was performed with both the Akaike information criterion (AIC) and the Bayesian information criterion (BIC). This selection was carried out using the step function in R with the full model, including all SNPs from the first stage and the three non-SNP covariates used as the initial model in the stepwise search and covariates allowed to be both deleted from and subsequently added back into the model (i.e., the direction argument of the step function was set to “both”).

We also performed LASSO bagging (bootstrap aggregation, or model resample averaging) [9]. This involved taking bootstrap samples of the data (sampling with replacement with sample size equal to that of the original data) and performing the first LASSO stage. We took 100 resamples and used the same *λ* parameter as for the original data for each resample. We were then able to calculate the resample model inclusion proportion (RMIP) for each SNP. The RMIP is a measure of how likely a given SNP is to be selected by the LASSO procedure if the data are perturbed.

The analysis was initially carried out without knowledge of the simulating model. We subsequently made comparison with this model.

## Results

The GAW17 data set contains 24,487 SNPs from 697 individuals. Filtering out SNPs with MAF < 0.01 leaves 6,356 SNPs. Retaining only one SNP from groups of identical SNPs left 6,321 SNPs to consider.

The phenotype simulating model included 39 SNPs in nine chromosomes, of which 7 SNPs from four chromosomes are common variants (MAF > 0.01). These SNPs are presented in Table 1.

**Table 1:** Common SNPs in model

SNPs with MAF > 0.01 in the simulating model for Q1. Gene	SNP	MAF	SNP effect size, *β*
*ARNT*	C1S6533	0.011478	0.56190
*FLT1*	C13S431	0.017217	0.74136
*FLT1*	C13S522	0.027977	0.61830
*FLT1*	C13S523	0.066714	0.64997
*HIF1A*	C14S1734	0.012195	0.21203
*KDR*	C4S1878	0.164993	0.13573
*KDR*	C4S1884	0.020803	0.29558

It is useful to check whether any of the SNPs in the simulating model are in high linkage disequilibrium (LD) with any other SNPs, because this may lead to SNPs showing up in the model selection in the place of the simulating SNPs. The simulating SNPs C1S6533, C14S1734, C4S1878, and C4S1884 are not in LD with any other SNP (pairwise *r*^2^ ≥ 0.1). The SNP in highest LD with C13S431 has a pairwise *r*^2^ = 0.315, but it is on a different chromosome. The SNPs C13S522 and C13S523 have their highest LD with each other (pairwise *r*^2^ = 0.142).

Our two-stage procedure was run with two different values of the LASSO tuning parameter *λ*, each obtained by using a 10-fold CV. The first parameter, termed *λ*_min_, minimizes the CV error. The second parameter, termed *λ*_1se_, is a stronger penalty to guard against model overfit and is the maximum value with its CV error within one standard error of the minimum CV error. Because the penalty parameter obtained by CV can vary depending on the random partitioning of observations, CV was repeated 10 times on replicate 1, and the median penalty parameter was used. This resulted in *λ*_min_ = 0.0914 and *λ*_1se_ = 0.160. (An alternative approach is to select *λ* so that a predetermined number of SNPs are selected; however, we approached the analysis without knowledge of how many SNPs were in the simulating model.)

As already mentioned, the second step was performed with the AIC and the BIC. The model selection procedure was performed on all 200 phenotype replicates. Table 2 summarizes the average number of times that the simulating model SNPs were selected.

**Table 2:** Mean number of times SNPs in simulating model were selected over 200 replicates

SNP	Lasso with *λ*_min_	+ AIC	+ BIC	Lasso with *λ*_1se_	+ AIC	+ BIC
C1S6533	0.445	0.440	0.365	0.040	0.040	0.040
C13S431	0.775	0.665	0.555	0.185	0.185	0.180
C13S522	0.985	0.970	0.880	0.850	0.850	0.850
C13S523	1.000	1.000	1.000	1.000	1.000	1.000
C14S1734	0.085	0.065	0.035	0.000	0.000	0.000
C4S1878	0.375	0.265	0.200	0.045	0.045	0.045
C4S1884	0.425	0.385	0.285	0.055	0.055	0.055
Total correct	4.09	3.79	3.32	2.175	2.175	2.170
Total selected	28.05	21.18	14.30	3.29	3.22	3.10

The mean number of total SNPs selected for each of the variations of the model selection procedure are also shown.

In Table 2 we see that if the LASSO procedure with *λ*_min_ is used, on average 28.1 SNPs are selected, of which 4.1 are in the simulating model. If stepwise selection with the BIC is also used, then on average 14.3 SNPs are selected, of which 3.3 are in the simulating model. So by applying the second step, we lose on average 0.8 SNP in the simulating model, but the proportion of selected SNPs in the simulating model is improved (from 14.6% to 23.2%). If stepwise selection with the AIC is used, then on average 21.2 SNPs are selected, of which 3.8 are in the simulating model.

We also see in Table 2 that if a LASSO procedure with *λ*_1se_ is used, then on average 3.3 SNPs are selected, of which 2.2 are in the simulating model. Subsequently applying stepwise selection with either the AIC or the BIC leads to little refinement of the model. This is because in this case we have used a stricter penalty in the LASSO step, so there are fewer SNPs to be potentially removed in the second step.

The determination of what is the best combination of the LASSO parameter *λ* and the type of subsequent stepwise selection (AIC or BIC) depends on the context. Use of *λ*_1se_ and the BIC is likely to result in identification of fewer spurious associations but also less true causal associations. If it is more important to ensure that all causal associations are captured for further investigation, then it may be better to use *λ*_min_ and the AIC.

After averaging over all 200 replicates, we found that after the LASSO step using *λ*_min_, six of the seven SNPs in the simulating model were selected more often than any other SNPs that were not in the simulating model. The seventh SNP in the simulating model (C14S1734) was the 21st most selected SNP (i.e., 14 SNPs not in the simulating model were selected more often). After applying stepwise selection with the BIC, C14S1734 was the 20th most selected SNP, and the other six SNPs in the simulating model were still the most selected. Note that there is a relationship between the mean number of times that SNPs in the simulating model are selected and the MAF and effect size of those SNPs (see Table 1). The three most often selected SNPs are those in the gene *FLT1* on chromosome 13. It appears to be no coincidence that these are the SNPs with the largest effect size. The two SNPs with the smallest effect size are C14S1734 (0.21203) and C4S1878 (0.13573). Although it is C4S1878 that has the smallest effect size, it is C14S1734 that is detected by the model selection procedure least often. It appears that this is due to C14S1734 having a much smaller MAF than C4S1878 (0.012 compared to 0.165).

### Stability analysis

**Figure 1:**
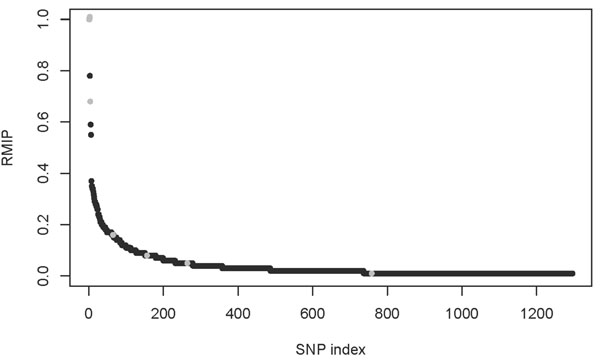
**Distribution of RMIP values for each SNP from LASSO bagging (resample model averaging)**. SNPs were ordered on the *x*-axis by RMIP value. Of the 6,321 SNPs, 1,296 had nonzero RMIP (zero values are not plotted). The SNPs used in the simulating model are plotted in gray.

We performed LASSO bagging with 100 resamples and fixed penalty parameter *λ* over all the resamples. The distribution of the RMIP values for each SNP is plotted in Figure 1. The SNPs in the simulating model are marked. The plot shows that only three of the seven SNPs in the simulating model have RMIP > 0.5. There are also three spurious associations detected with RMIP > 0.5. It is important to note that here we are considering only a single replicate (replicate 1), unlike earlier results, which were obtained by averaging over all 200 replicates. This is indicative of the problems of insufficient sample size, and the resultant tradeoff between false negatives and false positives.

## Discussion and conclusions

Simultaneous analysis of SNPs with penalized-regression approaches, such as the LASSO method, have gained attention recently. It is an open question as to what is the best sequence of steps when using these approaches. Here, we used the LASSO to identify a small set of SNPs that are then used as candidates in a standard model selection procedure. Recently, Cho et al. [3] proposed a procedure in which SNPs are initially filtered out based on single-SNP association before performing the penalized regression. Our investigations suggest that filtering on the basis of single-SNP association as a first step may leave out SNPs that would be selected by penalized regression.

We also note that model selection with the LASSO (using the glmnet package in R) is remarkably fast. The analysis of the GAW17 data for a single phenotype replicate can be carried out in a matter of seconds on a desktop computer. The glmnet package computation time scales linearly with the number of observations, the number of covariates, and the number of selected covariates, so it is quite feasible to analyze much larger data sets. We also experimented with the hyper-LASSO procedure introduced by Hoggart et al. [1]. This procedure is promising in terms of its model selection properties, but for large data sets the computation time is substantially greater than for the LASSO.

## Competing interests

The authors declare that they have no competing interests.

## Authors’ contributions

AJM, SG and SRW conceived the statistical analysis. AJM carried out the statistical analysis with assistance from CM. AJM drafted the manuscript. All authors read and approved the final manuscript.
